# *SPTBN2* Promotes the Progression of Thyroid Cancer by Accelerating G1/S Transition and Inhibiting Apoptosis

**DOI:** 10.1155/2022/2562595

**Published:** 2022-08-03

**Authors:** Xiaofen Zhou, Lizhi Lin, Yufeng Qi, Min Xu, Qiding Xu, Yinghao Wang, Jinmiao Qu

**Affiliations:** ^1^Department of Surgical Oncology, First Affiliated Hospital of Wenzhou Medical University, Wenzhou, 325000 Zhejiang, China; ^2^Department of Operating Theatre, The First Affiliated Hospital of Wenzhou Medical University, Wenzhou, Zhejiang 325000, China

## Abstract

**Background:**

Thyroid carcinoma (TC) is an increasingly common malignancy of endocrine organs, and its most frequently encountered histotype is papillary thyroid cancer (PTC). Identifying new potential gene alterations is important for completely elucidating the mechanism of PTC initiation and progression. Thus, we performed whole transcriptome sequence analysis (RNA-seq) on 79 PTC tissue samples and paired adjacent nontumor tissue samples to study the molecular mechanism of TC tumorigenesis and progression further. The results of RNA-seq analysis showed that spectrin beta, nonerythrocytic 2 (*SPTBN2*), was markedly overexpressed in PTC tissues relative to that in the paired nontumor tissues. Additionally, the analysis results for 502 PTC samples and 58 nontumor thyroid samples from The Cancer Genome Atlas dataset were consistent with our RNA-seq results. However, the molecular mechanisms and function of *SPTBN2* in TC progression remain unknown.

**Methods:**

We examined *SPTBN2* gene expression in 48 papillary thyroid tumor tissues and paired adjacent normal thyroid tissues by using qRT-PCR. *SPTBN2* expression in the TC cell lines was silenced by small interfering RNA. Then, the transfected TC cells were used to investigate the in vitro function of *SPTBN2*.

**Result:**

The expression of *SPTBN2* was significantly upregulated in our RNA-seq cohort, our local validated cohort, and TCGA RNA-seq cohort. The results of the in vitro experiment revealed that in TC cell lines, *SPTBN2* downregulation considerably suppressed tumor cell proliferation, the cell cycle, migration, colony formation, and invasion and induced cell apoptosis. Furthermore, the protein levels of CCNE2, CDK2, CDK4, and Bcl-2 were downregulated, and those of P21, Bax, cleaved caspase-8, and cleaved caspase-3 had increased in transfected TC cells relative to in control TC cells.

**Conclusion:**

The downregulation of *SPTBN2* caused apoptosis and retarded G1/S cell cycle transition in TC cells. Thus, *SPTBN2* may be a good candidate gene for TC diagnosis and therapy.

## 1. Introduction

Thyroid carcinoma (TC) is a highly common malignancy of endocrine organs; this disease accounted for approximately 2.9% of all new cases of malignancies in the United States of America in 2019 [1]. In the United States of America, the morbidity of thyroid cancer is now 2 times greater than that 30 years ago, and thyroid cancer is now the sixth most prevalent cancer in women [2, 3]. In the majority population, papillary thyroid cancer (PTC) is the most common subtype of TC and accounts for 85% of newly diagnosed TC cases [4]. Although the morbidity of thyroid cancer is high, PTC has a good prognosis with a >90% 10-year survival rate [5, 6]. However, a large number of patients with PTC experience metastasis and recurrence after conventional therapeutic strategies [7]. Therefore, further studies on the probable molecular mechanism of thyroid tumors are needed to provide early accurate diagnosis and appropriate treatment to patients with TC.

With the advent of whole transcriptome sequencing (RNA-seq) technology, next-generation sequencing has become widely utilized to identify changes in genes between tumors and normal tissues. Although numerous studies have been carried out to explore the probable mechanisms of thyroid tumors, the precise biomechanisms underlying the progression and occurrence of thyroid tumors remain to be elucidated fully. A growing number of reports have shown that genetic alterations, such as gene mutations, epigenetic modifications, and copy number variations, play an important role in the initiation of TC [8, 9]. For example, the point mutation of B-type Raf kinase can promote PTC tumorigenesis and progression via the initiation of the mitogen-activated protein kinase pathway [10, 11]. Furthermore, PAX8/PPAR*γ* rearrangements, RET/PTC rearrangements, and RAS mutations are important for the initiation and progression of differentiated TC [12–14]. Several biomarkers have been identified as appropriate diagnostic markers and potential therapeutic targets for TC. However, the identification of new potential gene alterations remains important for completely elucidating the mechanism of TC initiation and progression.

We performed RNA-seq on 79 PTC tissue samples and paired adjacent nontumor tissue samples to further study the underlying molecular mechanisms of TC. Through bioinformatics analysis, we discovered that spectrin beta, nonerythrocytic 2 (*SPTBN2*), a gene located on chromosome 11q13 in humans and encoding the membrane scaffold protein beta-III spectrin, is a potential powerful candidate gene that is associated with TC initiation and progression. Beta-III spectrin maintains dendritic architecture and stabilizes several membrane proteins [15]. A mutation in *SPTBN2* causes spinocerebellar ataxias type-5, a human neurodegenerative disorder that is characterized by progressive locomotor incoordination, slurred speech, and uncoordinated eye movements [16, 17]. Another study reported that the mutation of *SPTBN2* can disrupt Hippo signaling activity in follicle cells [18]. However, the previous study mainly focused on the effect of *SPTBN2* mutation on spinocerebellar ataxias type-5. The effect of the copy number variation of *SPTBN2* on the tumorigenesis and metastasis of malignancies has never been studied. Here, we were the first to prove that *SPTBN2* acts as a potential oncogene in the tumorigenesis and metastasis of thyroid malignancies by inhibiting apoptosis and promoting the G1/S cell cycle transition in malignant thyroid cells.

## 2. Materials and Methods

### 2.1. Tissues

The fresh malignant thyroid samples and paired nontumor thyroid samples that were used in this study were obtained from patients with PTC who underwent thyroid surgery at the First Affiliated Hospital of Wenzhou Medical University (China) from May 2017 to April 2019. The patients did not undergo chemo- or radiotherapy for pretreatment. All of the samples were snap-frozen in liquid nitrogen and stored at −80°C before RNA extraction. All of the tumor tissues used in the present study were examined blindly by three independent senior thyroid pathologists. Written consent/assent for the usage of the thyroid samples was given by the patients and their relatives. The procedures that were performed in our study were approved by and conducted in accordance with the ethical standards of the Institutional Review Board of the First Affiliated Hospital of Wenzhou Medical University (approval no. 2012-57).

### 2.2. The Cancer Genome Atlas Data

Data, including the expression profiles of the *SPTBN2* gene (502 PTC samples and 58 nontumor samples) with complete clinicopathological characteristics, were collected from The Cancer Genome Atlas (TCGA) databank for further analysis. All data related to the specimens were downloaded from TCGA website (https://tcgadata.nci.nih.gov/tcga).

### 2.3. Cell Culture

HTORI-3 (normal thyroid epithelial cells; RRID, CVCL 4W02) and KTC-1 (poorly differentiated thyroid cancer; RRID, CVCL 6300) were obtained from the Stem Cell Bank of the Chinese Academy of Sciences. TPC-1 (thyroid gland papillary carcinoma; RRID, CVCL 6298) and BCPAP (thyroid gland papillary carcinoma; RRID, CVCL 0153) were generously donated by Prof. Ming-Zhao Xing (School of Medicine, Johns Hopkins University, US). The cells were all incubated in Roswell Park Memorial Institute 1640 (RPMI-1640) medium (Invitrogen, Waltham, MA, US) containing 100 *μ*g/mL penicillin-streptomycin (Sigma Aldrich, US) and 10% fetal bovine serum (FBS; Invitrogen, Waltham, MA, US). The cells were kept in a humidified incubator (Thermo Fisher Scientific, Waltham, MA, US) at 37°C and 5% CO_2_.

### 2.4. Cell Transfection

Small interfering RNA (siRNA) for *SPTBN2* and the negative control siRNA for cell interference experiments were synthesized by Gene Pharma Co., Ltd (Gene Pharma, Shanghai, China) (Table 1). KTC-1 (8 × 10^4^ cells per well) and BCPAP (7 × 10^4^ cells per well) were plated on six well-plates and incubated for 24 h before transfection. siRNA was transfected into the TC cell lines by using Lipofectamine RNAiMAX Reagent (13778, Invitrogen, Carlsbad, CA, US). The transfected TC cells were harvested from the six-well plates at 48 h posttransfection, and knockdown efficacy was determined via qRT-PCR.

### 2.5. RNA Extraction and qRT-PCR Analysis

Total RNA was extracted from thyroid tissues or cell lines with TRIzol reagent (15596026, Invitrogen, US). Afterward, the A260/A280 ratio was used to evaluate RNA quality. By using a ReverTra Ace qPCR RT Kit (TOYOBO Life Science, Cat. No: FSQ-101, Japan), RNA was converted into cDNA. RNA expression levels were detected via qRT-PCR with an AB Prism 7500 fast system (Applied Biosystems, US) and SYBR qPCR Mix (QPS-201; TOYOBO Life Science, Japan). The primer pair details are shown in Table 2.

### 2.6. Cell Counting Kit-8 Proliferation Assay

The Cell Counting Kit-8 (CCK-8, C0038; Beyotime Institute of Biotechnology, China) assay was performed to evaluate cell proliferation capability. KTC-1 and BCPAP-1 (1.5 × 10^3^ cells/well in 96-well plates) transfected with Si-NC or *SPTBN2* siRNA were cultured in RPMI-1640 medium+10% FBS for 1–4 days. Afterward, CCK-8 solution (10 *μ*L) and RPMI-1640 medium (100 *μ*L) were added into each well at 24, 48, 72, and 96 h, and the samples were incubated at 37°C in 5% CO_2_ for 3 h. Then, the absorbance at 450 nm was determined.

### 2.7. Colony Formation Assay

The treated TC cells (1.5 × 10^4^ cells per well) were incubated in 10% FBS/RPMI-1640 medium in six-well plates for 7 days. Subsequently, the cells were washed three times by using phosphate-buffered saline (PBS) and fixed with 4% paraformaldehyde solution for 30 min. Lastly, all of the cells were rinsed with PBS three times and stained by using crystal violet (0.01%).

### 2.8. Migration and Invasion Assays

Migration capacity was detected via a Transwell migration assay. A Transwell cell culture chamber (8 *μ*m pore size, 3422, Corning, NY, US) was used for the in vitro Transwell migration assay. A total of 3.5 × 10^4^ transfected TC cells were suspended in 300 *μ*L of medium (RPMI-1640, free serum) and placed in the upper insert. The lower outer well contained 650 *μ*L of 10% serum RPMI-1640 medium. After 20 h of incubation, the nonmigrated cells on the upper side of each membrane were removed by using sterile cotton swabs, and the migrated cells on the lower surface were fixed in 4% paraformaldehyde solution and stained with crystal violet (0.01%). Cell invasion capacity was evaluated by using an invasion chamber (354480, Corning, NY, US). All of the steps in this procedure were the same as those followed in the Transwell migration assay.

### 2.9. Apoptosis Analysis

Cell apoptosis was measured by applying an Annexin V-fluorescein isothiocyanate/propidium iodide apoptosis detection kit (556547, BD, US). TC cells (5–6 × 10 cells per well) were cultured in six-well plates for 1 day and transfected for 2 days. The cells were collected and centrifuged at 1100 rpm for 5 min. Then, the supernatant was decanted, and the cells were washed with PBS (1 mL). These processes were repeated three times. Afterward, each tube was added with 4 *μ*L of Annexin V-FITC and 4 *μ*L of PI and then placed for 20 min in the dark. Apoptosis was studied with a BD FACSCalibur flow cytometer (Becton-Dickinson, CA, US).

### 2.10. Cell Cycle Assay

7-Amino-actinomycin D (7-AAD; 559925, BD Pharmingen, CA, US) was used for cell cycle analysis. Transfected TC cells (1 × 10^6^ cells per well) were collected from six-well plates and washed with PBS. Then, the TC cells were immersed in 70% ethanol overnight (4°C). Finally, the cells were rewashed with PBS and stained with 7-AAD for 10 min. The cell cycle assay was performed with a BD FACSCalibur flow cytometer (Becton-Dickinson, CA, US).

### 2.11. Gene Set Enrichment Analysis

Gene set enrichment analysis (GSEA) 4.0.2 software (http://www.broadinstitute.org/gsea) was used to evaluate the pathways that were associated with *SPTBN2* expression in TCGA dataset. For GSEA, PTC tissues (502) were divided equally into the high and low *SPTBN2* groups. The C2: curated gene sets (CP: KEGG gene set) used in GSEA were downloaded from the MSigDB database (version 7.1, https://www.gsea-msigdb.org/gsea/msigdb) [15, 16].

### 2.12. Western Blot Analysis

The protein was prepared from the cells by using RIPA lysis buffer. Protein concentrations were quantified by using a bicinchoninic acid assay kit (23227, Thermo Fisher Scientific). The total cell lysate proteins were separated by using SDS/PAGE gel (10%, 80–120 V) and electrotransferred onto a 0.22 *μ*m polyvinylidene fluoride membrane (PVDF; 300 mA for 70–100 min). Then, the PVDF membranes were incubated with 5% skimmed milk (232100, BD, MD, US) for 90 min at normal room temperature. Subsequently, the membranes were incubated with the primary antibody at 4°C overnight and incubated with the corresponding secondary antibody at room temperature for 1 h. The primary antibodies were as follows: SPTBN2 (1 : 500 dilution; human; 55107-1-AP, ProteinTech Group, Inc., China), Bax (1 : 2000 dilution; human; 50599-2-Ig, ProteinTech Group, Inc., China), caspase-8 (1 : 500 dilution; human; 13423-1-AP, ProteinTech Group, Inc., China), caspase-3 (1 : 500 dilution; human; 19677-1-AP, ProteinTech Group, Inc., China), Bcl-2 (1 : 500 dilution; human; ab182858, Abcam, Shanghai, China), CCNE2 (1 : 500 dilution; human; 507032, ZENBIO, Chengdu, China), CDK2 (1 : 500 dilution; human; 10122-1-AP, ProteinTech Group, Inc., China), CDK4 (1 : 500 dilution; human; 11026-1-AP, ProteinTech Group, Inc., China), and P21 (1 : 500 dilution; human; 10355-1-AP, ProteinTech Group, Inc., China).

### 2.13. Statistical Analysis

The differential expression of *SPTBN2* between TC and paired nontumor samples was analyzed through a *t*-test (2-tailed; paired *t*-test or independent *t*-test). Medcalc was used to perform receiver operating characteristic (ROC) analysis. The *χ*^2^ test and Fisher's exact test were used to evaluate the associations between clinicopathological characteristics and *SPTBN2* expression. Univariate and multivariate Cox regression analyses were conducted to evaluate the association between *SPTBN2* and tumor size. The analyses were performed with the statistical software SPSS (IBM SPSS, v23.0; SPSS Inc.) and Medcalc (v12.7.0; MedCalc Software, Ltd). *P* < 0.05 (∗) was considered statistically significant.

## 3. Results

### 3.1. *SPTBN2* Was Highly Overexpressed in PTC

**Figure 1:**
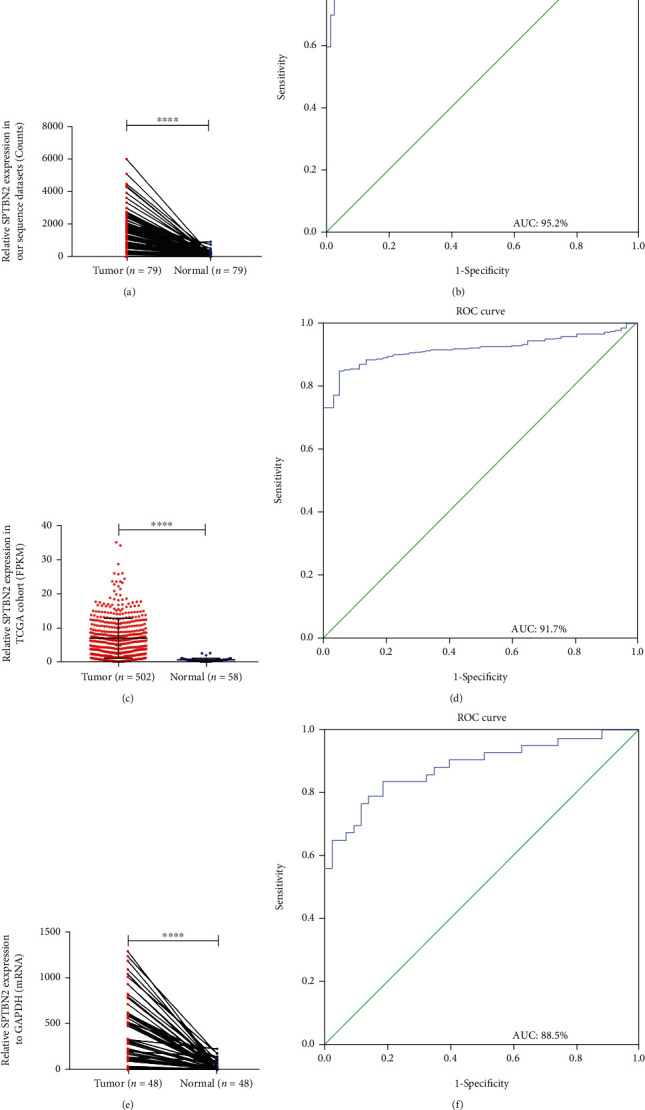
*SPTBN2* in PTC samples was highly upregulated compared with that in nontumor tissues. (a) RNA-seq dataset containing 79 PTC tissue samples and paired adjacent nontumor tissue samples (*P* < 0.0001). (b) ROC for the expression of *SPTBN2* in the RNA-seq cohort (AUC = 95.2%, sensitivity = 89.9%, and specificity = 2.4%) (*P* < 0.001). (c) TCGA dataset containing 502 PTC tissues and 58 normal thyroid tissues (*P* < 0.0001). (d) ROC for the expression of *SPTBN2* in TCGA cohort (AUC = 91.7%, sensitivity = 85.1%, and specificity = 94.8%) (*P* < 0.001). (e) Local validated cohort containing 48 PTC tissue samples and paired adjacent nontumor tissue samples (*P* < 0.0001). (f) ROC for the expression of *SPTBN2* in the local validated cohort (AUC = 88.5%, sensitivity = 83.7%, and specificity = 81.4%) (*P* < 0.001). The optimal cutoff value of *SPTBN2* expression was determined by maximizing the Youden index value. Youden index = (sensitivity + specificity) − 1. The data are presented as the mean ± standard deviation (three independent experiments). ^∗∗∗∗^*P* < 0.0001.

RNA-seq was performed on 79 PTC tissue samples and paired adjacent nontumor tissues to examine the potential mechanism of PTC. We found that the expression of *SPTBN2* in the PTC samples was highly upregulated compared with that in the adjacent nontumor thyroid tissues (Figure 1(a), Supplementary Table 1). Tissues from patients with PTC and normal thyroid tissues in TCGA dataset were also investigated (Figure 1(c)). The mRNA expression level of *SPTBN2* was also upregulated in our local validated cohort (48 PTC patients) as revealed by qRT-PCR (Figure 1(e)). Both results indicated that *SPTBN2* was highly upregulated in the PTC samples relative to that in the nontumor thyroid samples. The diagnostic potential of *SPTBN2* was evaluated on the basis of a ROC curve. ROC curve analysis was performed on all of the above cohorts (our RNA-seq cohort, our local validated cohort, and TCGA RNA-seq cohort) (Figures 1(b), 1(d), and 1(f)). The results indicated that *SPTBN2* is a potential gene for the diagnosis of PTC.

### 3.2. *SPTBN2* Expression Was Closely Related to Clinicopathological Characteristics in PTC

We examined the relationship between clinicopathological features and *SPTBN2* expression in our local validated cohort and TCGA cohort to gain insights into the mechanism of *SPTBN2* in the progression of PTC. The PTC tissues in TCGA cohort (502 patients with PTC) and our local validated cohort (48 patients with PTC) were classified individually into the high *SPTBN2* expression group or the low *SPTBN2* expression group in accordance with their median expression level value of *SPTBN2*. The results of the analyses showed that in TCGA cohort, histological type, LNM, tumor size, and disease stage were related to *SPTBN2* upregulation (Table 3). Similar results were found for our validated cohort. Specifically, the upregulation of *SPTBN2* was correlated with tumor size (*P* = 0.004), disease stage (*P* < 0.001), and LNM (*P* = 0.002) (Table 4). We omit the nonstatistically significant clinicopathologic features (Table 3) in Table 4. All analytical results provided support that *SPTBN2* may act as an oncogene in PTC.

### 3.3. *SPTBN2* Overexpression Was Correlated with Increased Tumor Size

Logistic regression studies were applied to evaluate the relationship between *SPTBN2* and tumor size in PTC. In the univariate logistic regression analysis, age, gender, *SPTBN2* expression, LNM, and disease stage were significant risk factors for increased tumor size (Table 5). Subsequently, multivariate logistic regression analysis was conducted on the variables that were found to be statistically significant in the univariate logistic regression analysis. The results indicated that age, gender, *SPTBN2* expression, and disease stage were significant independent risk factors for increased tumor size (Table 6).

### 3.4. Effects of the Underexpression of *SPTBN2* on the Proliferation Capacity of TC Cell Lines

**Figure 2:**
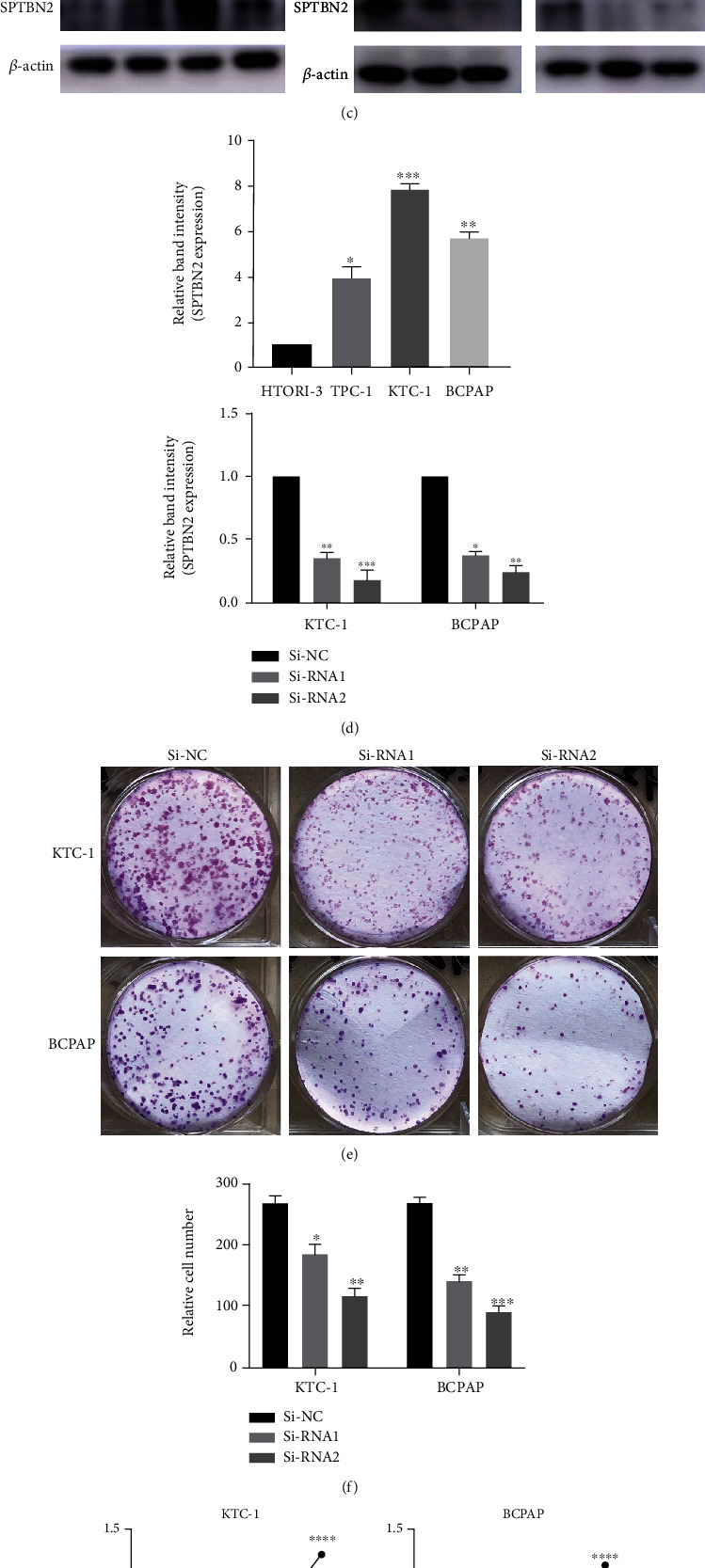
*SPTBN2* expression in thyroid cells and siRNA transfection efficiency in KTC-1 and BCPAP. *SPTBN2* knockdown inhibited TC cell line proliferation. (a) Relative expression of *SPTBN2* (normalized to GADPH) in the three malignant thyroid cell lines and HTORI-3 (normal thyroid follicular epithelial cell line). (b) Relative expression of *SPTBN2* (normalized to GADPH) in the TC cell lines (KTC-1 and the BCPAP) after siRNA treatment as detected via qRT-PCR. (c) *SPTBN2* protein level in the thyroid cell lines HTORI-3, TPC-1, KTC-1, and BCPAP and the siRNA-transfected KTC-1 and BCPAP cell lines as determined via WB analysis. (d) Quantitative results of the (C) WB analysis (normalized to *β*-actin). (e) Colony formation assay. TC cells treated with siRNA were plated onto six-well plates and incubated for 7 days. Compared with that of the Si-NC group, the proliferation capacity of the malignant thyroid cell lines in the Si-RNA1 and Si-RNA2 groups was exceedingly high. (f) Relative quantification of the (E) colony formation assay. (g) CCK-8 proliferation assay was conducted to quantify the proliferation of transfected cells. The data are presented as the mean ± standard deviation (three independent experiments). ^∗^*P* < 0.05;  ^∗∗^*P* < 0.01;  ^∗∗∗^*P* < 0.001;  ^∗∗∗∗^*P* < 0.0001.

We evaluated the RNA and protein expression levels of *SPTBN2* in three malignant thyroid cell lines and HTORI-3. As shown in Figures 2(a) and 2(c), the mRNA expression level of *SPTBN2* in the malignant cell lines was markedly greater than that in the normal cell lines (HTORI-3). Clearly, *SPTBN2* was commonly overexpressed in thyroid malignancy tissues and cell lines. siRNA was used to knock down *SPTBN2* in malignant thyroid cell lines (BCPAP and KTC-1) with high *SPTBN2* expression to further study the function of *SPTBN2* in thyroid malignancies. The transfection efficiency of the siRNA was investigated by using qRT-PCR and Western blot (WB) analyses. The results indicated that the siRNA transfection efficiency exceeded 50% (Figures 2(b) and 2(d)). Then, the proliferation capacity of the malignant thyroid cells was investigated by using CCK-8 and colony formation assays to evaluate the function of *SPTBN2* in TC. The results of the CCK-8 and colony formation assays showed that the downregulation of *SPTBN2* markedly suppressed the proliferation of the KTC-1 and BCPAP cell lines (Figures 2(e)–2(g)).

### 3.5. Knockdown of *SPTBN2* Inhibited TC Cell Migration and Invasion In Vitro

**Figure 3:**
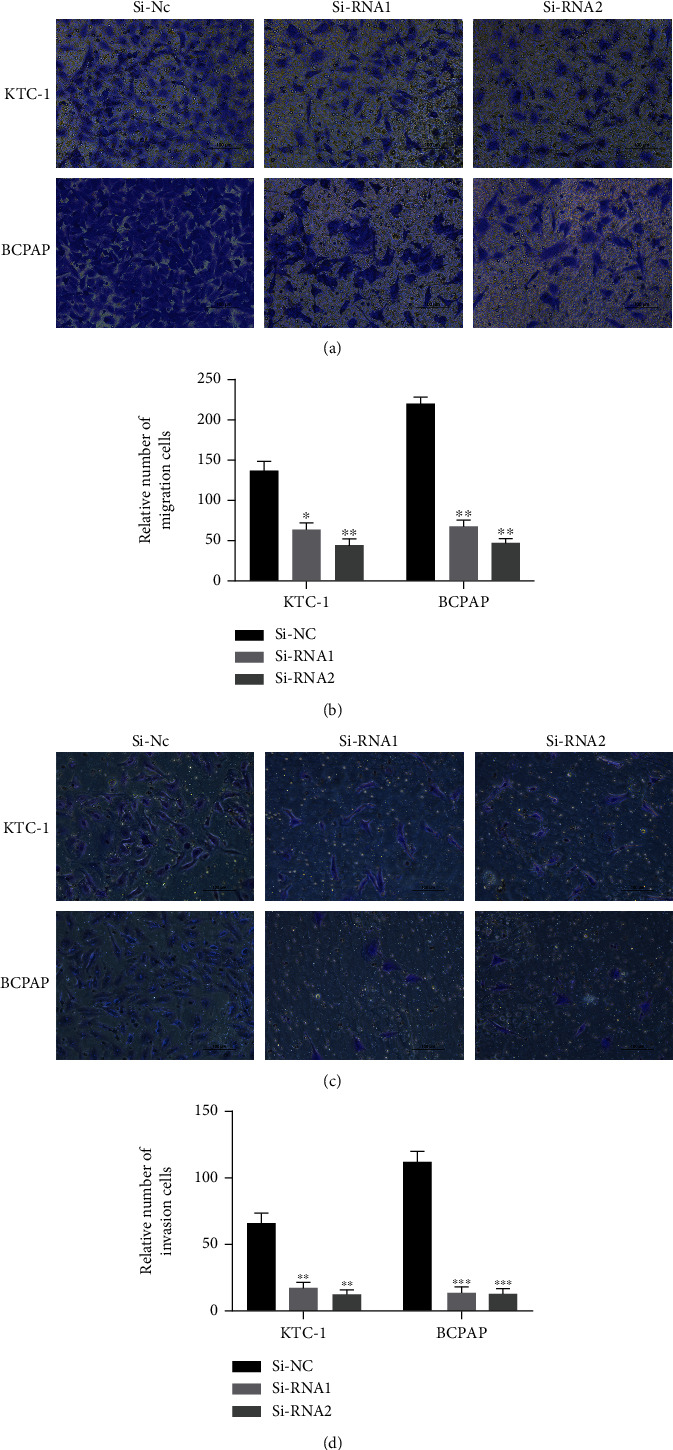
Underexpression of *SPTBN2* suppressed cell migration and invasion capability in malignant thyroid cell lines (KTC-1 and BCPAP). (a) Cell migration assays. The migration capability of TC cells was suppressed by the downregulation of *SPTBN2* (compared with Si-NC). (b) Quantitative results of the cell migration assay. (c) Cell invasion assays. The invasion capability of TC cells was inhibited by the knockdown of *SPTBN2* (compared with Si-NC). (d) Quantitative results of the cell invasion assay. The data are presented the mean ± standard deviation (three independent experiments). ^∗^*P* < 0.05;  ^∗∗^*P* < 0.01;  ^∗∗∗^*P* < 0.001.

By subjecting TCGA cohort and our validated cohort to the *χ*^2^ test, we found that LNM was significantly correlated with *SPTBN2* expression (Tables 3 and 4). Therefore, we studied the function of *SPTBN2* in the migration/invasion capacities of TC. Migration capacity was assessed by using migration assays. The results demonstrated that in malignant thyroid cell lines, migration capacity was significantly suppressed by the knockdown of *SPTBN2* (Figures 3(a) and 3(b)). The invasion capacity of the transfected TC cell lines was detected via invasion assays. The results showed that the invasion capability of the TC cell lines was also inhibited by the knockdown of *SPTBN2* (Figures 3(c) and 3(d)).

### 3.6. Knockdown of *SPTBN2* Induced Apoptosis and Retarded G1/S Cell Cycle Transition in TC Cell Lines In Vitro

**Figure 4:**
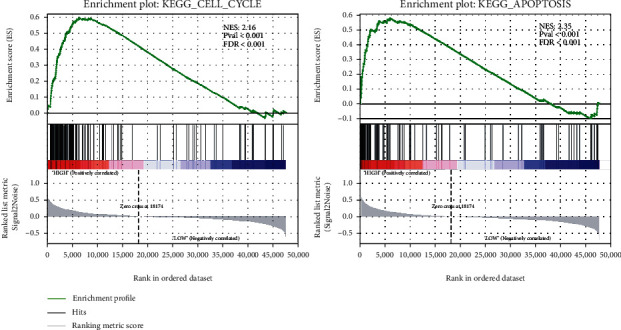
GSEA was used to evaluate the pathways that were associated with SPTBN2 expression in TCGA dataset.

**Figure 5:**
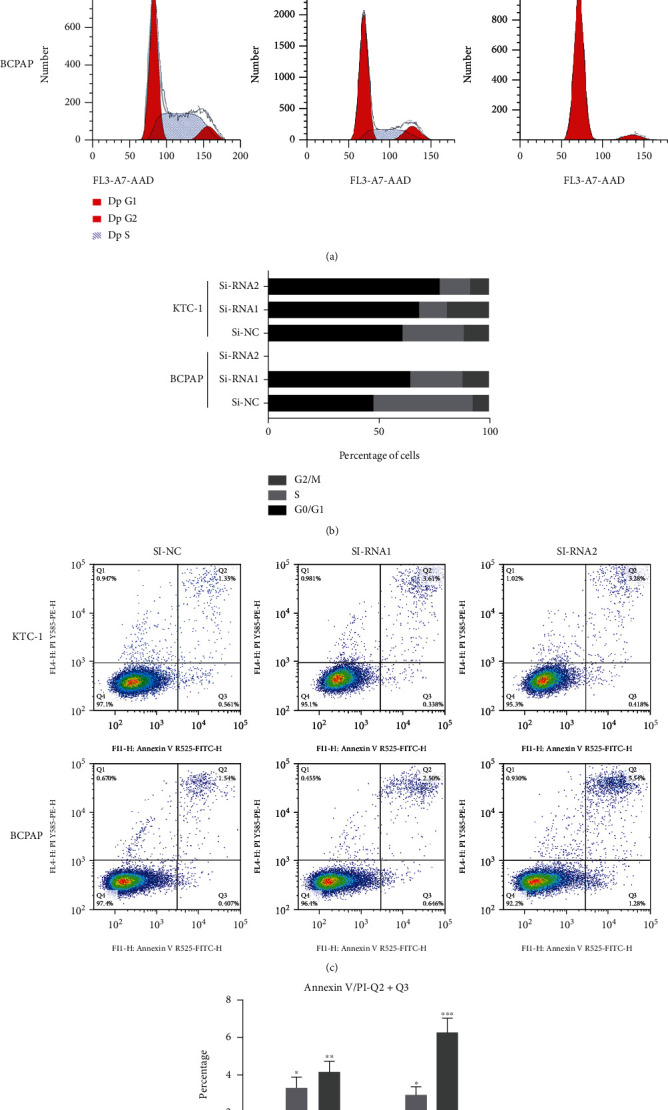
*SPTBN2* gene knockdown induced apoptosis and retarded G1/S cell cycle transition in TC cells. (a) Flow cytometry assay of cell cycle distribution after *SPTBN2* downregulation. (b) Distribution of cell cycle stages. (c) Flow cytometry analysis revealed the apoptosis rates of siRNA-transfected KTC-1 and BCPAP cells. (d) Quantitative analysis of apoptotic cell percentages. Percentage of cell apoptosis = Q2 (early apoptosis) + Q3 (late apoptosis). ^∗^*P* < 0.05,  ^∗∗^*P* < 0.01, and^∗∗∗^*P* < 0.001. The data are presented as the mean ± standard deviation (three independent experiments).

The GSEA results revealed that in TCGA cohort, the cell cycle/apoptosis pathway was closely associated with *SPTBN2* expression in thyroid cancer (Figure 4). Given that cell cycle regulation is closely associated with cell proliferation, we analyzed the function of *SPTBN2* in the TC cell cycle. The proportion of G1-phase cells increased with the knockdown of *SPTBN2*, whereas that of S-phase KTC-1 and BCPAP cells decreased (Figures 5(a) and 5(b)). Hence, the G1/S transition was significantly suppressed with the knockdown of *SPTBN2*. Cell apoptosis is another cause of cell growth inhibition. Therefore, the percentage of apoptotic cells after siRNA transfection was assessed. The ratio of apoptotic cells after transfection with *SPTBN2*-siRNA significantly increased relative to that after transfection with the negative control (Figures 5(c) and 5(d)). These results indicated that *SPTBN2* markedly promoted cell cycle transition and suppressed TC cell apoptosis.

### 3.7. *SPTBN2* Knockdown Suppressed the Proliferation of PTC Cells by Arresting G1/S Transition and Promoting Cell Apoptosis

**Figure 6:**
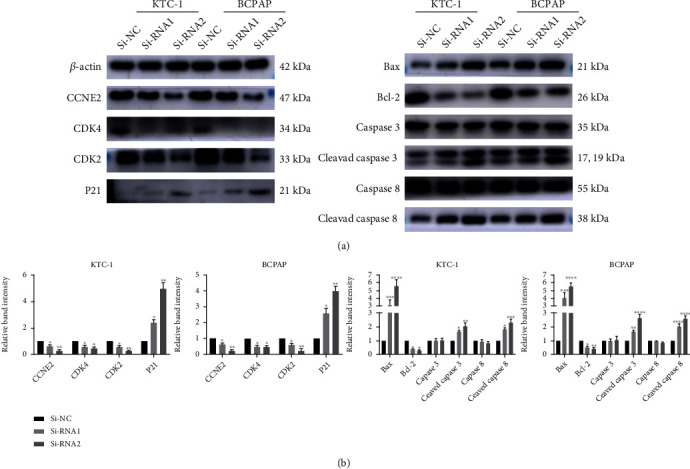
Downregulation of *SPTBN2* inhibited the tumorigenesis and progression of TC cell lines by arresting G1/S transition and promoting cell apoptosis. (a) Levels of CCNE2, CDK2, CDK4, Bcl-2, P21, caspase-3, cleaved-caspase3, caspase-8, cleaved-caspase8, and Bax were evaluated via WB analysis with *β*-actin as the loading control. (b) Quantitative results of WB analysis (normalized to *β*-actin). ^∗^*P* < 0.05,  ^∗∗^*P* < 0.01,  ^∗∗∗^*P* < 0.001, and^∗∗∗∗^*P* < 0.0001. The data are presented as the mean ± standard deviation (three independent experiments).

After transfecting TC cells with siRNA, we performed WB analysis to detect the expression levels of signaling biomarker proteins that were correlated with the cell cycle/apoptosis in TC cell lines. The results illustrated that in TC cells, the knockdown of *SPTBN2* significantly inhibited the expression levels of cell cycle/anti-apoptotic proteins, including CCNE2, CDK2, CDK4, and Bcl-2 (Figure 6(a)). Furthermore, the downregulation of *SPTBN2* significantly increased the expression levels of P21, cleaved caspase-8, cleaved caspase-3, and Bax in TC cells (Figures 6(a) and 6(b)). These results indicated that the knockdown of *SPTBN2* inhibited the G1/S phase transition via the upregulation of P21 and the downregulation of CCNE2, CDK2, and CDK4. Moreover, it promoted TC cell apoptosis via the upregulation of Bax, cleaved-caspase-3, and cleaved-caspase-8 and the downregulation of Bcl-2.

## 4. Discussion

Thyroid cancer is a heavy burden on society, and its morbidity has continuously increased on an annual basis [17]. PTC, the most frequent thyroid cancer type (>85%), has been attracting growing attention [18]. Approximately 10% of patients with PTC cannot benefit from conventional therapies due to metastasis and recurrence [19]. Thus, exploring the fundamental molecular mechanisms of TC is essential for the identification and management of this malignancy. A number of studies have reported that numerous genes play a vital role in the tumorigenesis of TC [20–22]. However, the current understanding of the molecular mechanism of TC is far from comprehensive.

The widely used next-generation sequencing technology provides an opportunity for elucidating the molecular mechanisms of TC. Previously, we subjected 79 paired PTC samples and nontumor thyroid tissues to whole transcriptome sequencing. Through RNA-seq data analysis, we discovered that *SPTBN2* expression in PTC samples was highly upregulated compared with that in adjacent nontumor thyroid samples. Furthermore, in patients with PTC in TCGA cohort and our local validated cohort (48 PTC patients), clinicopathological characteristics (tumor size, LNM, and disease stage) showed a high positive correlation with the high expression of *SPTBN2*.

The *SPTBN2* gene is located on chromosome 11q13.2 [23]. It codes for a protein called *SPTBN2* or beta-III spectrin. Beta-III spectrin functions in diverse cellular functions, including the determination of cell form, the arrangement of transmembrane proteins, and the association of organelles. *SPTBN2* mutation can result in spinocerebellar ataxias type-5. This disease is characterized by neural degeneration, uncoordinated eye movements, and locomotor incoordination. SPTBN1, which is an important paralog of *SPTBN2*, and other nonerythroid spectrins exert either oncogenic or tumor suppressor activities by affecting various cancer-related biological processes, including cell differentiation, DNA damage repair, and epithelial-to-mesenchymal transition [24]. A growing body of evidence has extensively proven that *SPTBN2* plays a vital role in the diagnosis of cholangiocarcinoma. For example, by using a bioinformatics approach, Feng et al. demonstrated that *SPTBN2* is highly associated with poor prognosis in ovarian cancer [25]. Zhang et al. and Yue et al. reported that *SPTBN2* may play a vital role in the progression of colorectal cancer [26, 27]. Wu et al. reported that *SPTBN2* is positively correlated with the poor prognosis of lung adenocarcinoma [28]. However, the probable mechanisms of *SPTBN2* in this malignancy has never been investigated before [29–31].

Although bioinformatics studies have revealed that *SPTBN2* may be correlated with the initiation and progression of malignancies, the molecular mechanism of *SPTBN2* in thyroid cancer has never been investigated. In this study, we discovered that *SPTBN2* was exceedingly overexpressed in PTC tissues in our RNA-seq and TCGA datasets. Then, we evaluated the expression of *SPTBN2* in 48 PTC samples and paired adjacent nontumor tissues via qRT-PCR and explored the biological function of *SPTBN2* in vitro. The results of qRT-PCR were consistent with those of previous RNA-seq and TCGA analyses.

Previous in vitro studies on TC cell lines (BCPAP and KTC-1) knocked down *SPTBN2* expression by using siRNA. The results of the cell experiments indicated that the knockdown of *SPTBN2* could inhibit proliferation and metastasis capacity and induce apoptosis in the TC cell line in vitro. Furthermore, the signaling pathways that might be associated with *SPTBN2* were analyzed via the GSEA. Since the GSEA showed downregulation of *SPTBN2* related to the apoptosis and cell cycle signaling pathways in TCGA dataset, we focused on the expression level of the apoptosis relative protein (Bax, Bcl-2, caspase-3, caspase-8, caspase-9, cleaved caspase-3, cleaved caspase-8, and cleaved caspase-9) and the cell-cycle relative protein (P21, CCNE2, CDK2, and CDK4) in the transfected TC cell line. Moreover, the downregulation of *SPTBN2* led to a substantial increase in the protein levels of Bax, cleaved-caspase-8, and cleaved-caspase-3 and a reduction in CCNE2, CDK2, CDK4, and Bcl-2. This discovery suggested that *SPTBN2* could promote the progression of TC cell lines by inhibiting G1/S transition and promoting apoptosis.

We showed that *SPTBN2* was closely associated with tumor size, LNM, and disease stage. Furthermore, in TC cells, the knockdown of *SPTBN2* inhibited G1/S transition and promoted apoptosis. These findings proved that *SPTBN2* is a good candidate gene for TC diagnosis, treatment, and prevention.

## Figures and Tables

**Table 1:** siRNA targeting human *SPTBN2.*

si-RNA name	Sequence (5′ to 3′)	
	Sense	Antisense
*SPTBN2* Si-RNA1	GCCAGUACAGUGACAUCAATT	UUGAUGUCACUGUACUGGCTT
*SPTBN2* Si-RNA2	GCUGCUGAUGCUGCCAUUATT	UAAUGGCAGCAUCAGCAGCTT

**Table 2:** Real-time PCR primers.

Primer name	Sequence (5′ to 3′)
*SPTBN2*-F	GGGCATCCTTGGCTGACT
*SPTBN2*-R	GACGGAAACCACCGACTG
GADPH-F	GGTCGGAGTCAACGGATTTG
GADPH-R	ATGAGCCCCAGCCTTCTCCAT

**Table 3:** The association between *SPTBN2* expression and clinicopathologic features in TCGA cohort.

Clinicopathologic features	High expression (*n* = 251)	Low expression (*n* = 251)	*X* ^2^	*P* value
Gender			0.253	0.615
Female	186	181		
Male	65	70		
Age (years)				
Mean ± SD	46.876 ± 15.236	47.809 ± 16.363	0.128	0.721
≤45	116	120		
>45	135	131		
Multi-nodularity			0.805	0.37
Yes	118	108		
No	133	143		
Histological type			26.605	<0.001^a^
Classical	202	149		
Other types	49	102		
Tumor size (mm)			18.594	<0.001^a^
≥20	120	73		
<20	131	178		
Lymph-node metastasis			27.167	<0.001^a^
Yes	140	82		
No	111	169		
Distant metastasis			0	1
Yes	5	4		
No	246	247		
Disease stage (AJCC7)			15.084	<0.001^a^
I+II	147	188		
III+IV	104	63		

Chi-square test. ^a^*P* value < 0.05. AJCC: American Joint Committee on Cancer.

**Table 4:** The association between *SPTBN2* expression and clinicopathologic features in the local validated cohort.

Clinicopathologic features	High expression (*n* = 24)	Low expression (*n* = 24)	*X* ^2^	*P* value
Tumor size (mm)			2.064	0.004^a^
≥20	18	8		
<20	6	16		
Lymph-node metastasis			12.343	<0.001^a^
Yes	20	8		
No	4	16		
Disease stage (AJCC7)			5.371	0.020^a^
I+II	9	17		
III+IV	15	7		

Chi-square test. ^a^*P* value < 0.05. AJCC: American Joint Committee on Cancer.

**Table 5:** Univariate logistic regression analysis for the risk of increased tumor size.

Factor	OR	95% CI	*P* value
Age (≤45 vs. >45)	1.969	1.362-2.845	<0.001^a^
Gender (male vs. female)	0.553	0.371-0.825	0.004^a^
*SPTBN2* expression (low vs. high.)	2.234	1.546-3.228	<0.001^a^
Histological type (PTC vs. others.)	0.963	0.651-1.424	0.85
Lymph-node metastasis	2.686	1.855-3.889	<0.001^a^
Disease stage (AJCC7)	11.117	7.196-17.199	<0.001^a^

Chi-square test. ^a^*P* value < 0.05. AJCC: American Joint Committee on Cancer.

**Table 6:** Multivariate logistic regression analysis for the risk of increased tumor size.

Factor	OR	95% CI	*P* value
Age (≤45 vs. >45)	0.127	0.050-0.326	<0.001^a^
Gender (male vs. female)	0.611	0.376-0.993	0.047^a^
*SPTBN2* expression (low vs. high)	1.664	1.071-2.583	0.023^a^
Disease stage (AJCC7)	51.751	19.838-135.004	<0.001^a^

Chi-square test. ^a^*P* value < 0.05. AJCC: American Joint Committee on Cancer.

## Data Availability

The datasets generated and/or analyzed during the current study are not publically available due to restrictions on data sharing imposed by the funding body but are available from the corresponding author upon reasonable request.

## Supplementary Materials

Supplementary Materials Comparison of *SPTBN2* mRNA expression between thyroid tumors and normal thyroid tissues in our local RNA-seq dataset.
