# Supplementary material for: SPTBN2 Promotes the Progression of Thyroid Cancer by Accelerating G1/S Transition and Inhibiting Apoptosis
Source: Dis Markers. 2022 Aug 3;2022:2562595. doi: 10.1155/2022/2562595 (PMC9365581; doi:10.1155/2022/2562595)
Supplement: Supplementary Materials — Comparison of SPTBN2 mRNA expression between thyroid tumors and normal thyroid tissues in our local RNA-seq dataset. [file 2562595.f1.docx]

|  | Supplementary Table I. The expression of SPTBN2 gene in 79 cases of thyroid papillary carcinoma compare with normal tissue by whole transcriptome sequencing. | | | | |
| --- | --- | --- | --- | --- | --- |
|  | Sample | RN-counts | RT-counts | Log 2 ratio (RT/RN) | RT/RN |
| 1 | 548811 | 87 | 616 | 2.823843045 | UP |
| 2 | 1152603 | 75 | 1496 | 4.318075769 | UP |
| 3 | 1153453 | 107 | 1712 | 4 | UP |
| 4 | 1253638 | 97 | 2562 | 4.723141918 | UP |
| 5 | 1267375 | 33 | 5116 | 7.27640643 | UP |
| 6 | 1267409 | 91 | 402 | 2.143257051 | UP |
| 7 | 1270716 | 132 | 0 | No Value | DOWN |
| 8 | 1272241 | 198 | 336 | 0.762960803 | UP |
| 9 | 1272253 | 68 | 4360 | 6.002649578 | UP |
| 10 | 1281991 | 276 | 271 | -0.026375415 | DOWN |
| 11 | 1281995 | 124 | 1073 | 3.11323805 | UP |
| 12 | 1283215 | 102 | 3017 | 4.886473639 | UP |
| 13 | 1284199 | 138 | 6016 | 5.446064395 | UP |
| 14 | 1287396 | 87 | 672 | 2.949373927 | UP |
| 15 | 1291231 | 267 | 736 | 1.462866024 | UP |
| 16 | 1292042 | 145 | 3013 | 4.377075868 | UP |
| 17 | 1295541 | 208 | 1726 | 3.052777031 | UP |
| 18 | 1295762 | 153 | 3631 | 4.568763372 | UP |
| 19 | 1297226 | 158 | 3940 | 4.640199166 | UP |
| 20 | 1302325 | 295 | 743 | 1.332647256 | UP |
| 21 | 1309208 | 182 | 1161 | 2.673357617 | UP |
| 22 | 1316314 | 130 | 1801 | 3.792214653 | UP |
| 23 | 1317299 | 80 | 1818 | 4.506208389 | UP |
| 24 | 1318404 | 43 | 52 | 0.274174963 | UP |
| 25 | 1320021 | 78 | 1967 | 4.656379023 | UP |
| 26 | 1326980 | 527 | 2544 | 2.271223804 | UP |
| 27 | 1327632 | 946 | 692 | -0.451068146 | DOWN |
| 28 | 1329390 | 182 | 784 | 2.106915204 | UP |
| 29 | 1331954 | 114 | 1311 | 3.523561956 | UP |
| 30 | 1335161 | 209 | 2761 | 3.723616041 | UP |
| 31 | 1335945 | 163 | 244 | 0.582009183 | UP |
| 32 | 1338955 | 99 | 2782 | 4.812550084 | UP |
| 33 | 1341742 | 125 | 543 | 2.119024103 | UP |
| 34 | 1342937 | 90 | 2751 | 4.933887328 | UP |
| 35 | 1344535 | 82 | 1364 | 4.056075924 | UP |
| 36 | 1345729 | 149 | 1139 | 2.934383511 | UP |
| 37 | 1348236 | 317 | 4494 | 3.825445379 | UP |
| 38 | 1350077 | 74 | 1318 | 4.154681289 | UP |
| 39 | 1353199 | 108 | 1933 | 4.16173842 | UP |
| 40 | 1356177 | 65 | 586 | 3.172389041 | UP |
| 41 | 1359874 | 150 | 785 | 2.387730153 | UP |
| 42 | 1360052 | 210 | 2113 | 3.330831534 | UP |
| 43 | 1363999 | 70 | 867 | 3.630605166 | UP |
| 44 | 1364219 | 122 | 560 | 2.198545679 | UP |
| 45 | 1365307 | 132 | 1614 | 3.612030744 | UP |
| 46 | 1365657 | 289 | 216 | -0.42003818 | DOWN |
| 47 | 1366593 | 154 | 754 | 2.291634173 | UP |
| 48 | 1368574 | 98 | 932 | 3.249476301 | UP |
| 49 | 1370585 | 49 | 1653 | 5.076161165 | UP |
| 50 | 1372096 | 147 | 840 | 2.514573173 | UP |
| 51 | 1374256 | 76 | 172 | 1.178337241 | UP |
| 52 | 1374809 | 28 | 2105 | 6.232249596 | UP |
| 53 | 1375934 | 404 | 2238 | 2.469782838 | UP |
| 54 | 1376040 | 153 | 2423 | 3.985190847 | UP |
| 55 | 1376519 | 66 | 682 | 3.36923381 | UP |
| 56 | 1382525 | 174 | 660 | 1.923378718 | UP |
| 57 | 1382636 | 79 | 2533 | 5.002850614 | UP |
| 58 | 1385076 | 97 | 4304 | 5.47154952 | UP |
| 59 | 1385097 | 78 | 864 | 3.469485283 | UP |
| 60 | 1391191 | 244 | 861 | 1.81913209 | UP |
| 61 | 1392926 | 240 | 3341 | 3.799173672 | UP |
| 62 | 1397219 | 201 | 196 | -0.036341847 | DOWN |
| 63 | 1401189 | 351 | 204 | -0.782901878 | DOWN |
| 64 | 1401703 | 56 | 1351 | 4.592457037 | UP |
| 65 | 1402293 | 212 | 316 | 0.575860294 | UP |
| 66 | 1404580 | 86 | 2679 | 4.961214111 | UP |
| 67 | 1406830 | 109 | 1815 | 4.057569508 | UP |
| 68 | 1411217 | 78 | 1862 | 4.577235139 | UP |
| 69 | 1411427 | 189 | 1800 | 3.251538767 | UP |
| 70 | 1411753 | 770 | 2018 | 1.389995824 | UP |
| 71 | 1412603 | 103 | 1998 | 4.277840341 | UP |
| 72 | 1412613 | 102 | 2286 | 4.486184346 | UP |
| 73 | 1414100 | 177 | 1546 | 3.126719054 | UP |
| 74 | 1417564 | 106 | 394 | 1.894131365 | UP |
| 75 | 1418404 | 114 | 1217 | 3.416223439 | UP |
| 76 | 1418978 | 222 | 1821 | 3.036099341 | UP |
| 77 | 1421725 | 261 | 720 | 1.4639471 | UP |
| 78 | 1426566 | 116 | 805 | 2.794863978 | UP |
| 79 | 1430045 | 152 | 2438 | 4.003554897 | UP |
|  | Abbreviations: RN , RNA normal tissues; RT, RNA tumor tissues; Counts, | | | | |
